# Comparative Analysis of the Postoperative Analgesic Effect of Caudal Ropivacaine Versus Ropivacaine With Nalbuphine in Infra-Umbilical Pediatric Surgeries: A Randomized, Double-Blind Pilot Study

**DOI:** 10.7759/cureus.76311

**Published:** 2024-12-24

**Authors:** G Sai Mahitha, Subrata K Singha, Mayank Kumar, Nitinkumar Borkar

**Affiliations:** 1 Anesthesiology and Critical Care, All India Institute of Medical Sciences, Raipur, Raipur, IND; 2 Pediatric Surgery, All India Institute of Medical Sciences, Raipur, Raipur, IND

**Keywords:** 0.75% ropivacine, caudal epidural analgesia, flacc scale, infraumbilical surgeries, nalbuphine

## Abstract

Postoperative pain in children leads to an immense stress response than adults, leading to an increased hospital stay and “pain memory.” Caudal epidural anesthesia is one of the most reliable, popular, and safe techniques that provide proper analgesia for infra-umbilical surgeries. A combination of local anesthetics and opioids reduces the dose-related adverse effects of each drug independently. In this study, we evaluated the drug nalbuphine as an additive to ropivacaine and plain ropivacaine in a caudal epidural to test the duration of postoperative analgesia and the need for IV paracetamol.

The study conducted was a prospective, randomized, double-blind, and pilot study with size of 60 children aged two to 12 years of American Society of Anesthesiologists (ASA) 1 and 2 posted for infra umbilical surgeries, divided into two groups of Group R (0.75 mL/kg of 0.25% ropivacaine) and Group RN (0.75 mL/kg of 0.25% ropivacaine with 0.1 mg/kg nalbuphine) with 30 each. A caudal epidural was administered 20 minutes before the incision after induction of anesthesia.

The child was monitored perioperatively for 24 hours for vitals, Face, Legs, Activity, Cry, and Consolability (FLACC) score, adverse effects, and the need for rescue analgesia, which were recorded and analyzed.

The addition of nalbuphine to caudal ropivacaine prolonged the analgesia from six hours to 12-18 hours postoperatively. It also decreased the doses of intravenous (IV) paracetamol and completely nil use in 50% of the population.

To conclude, nalbuphine as an additive in caudal epidural compared to plain ropivacaine alone provided better analgesia. It prolonged the duration of analgesia with decreased use of postoperative IV paracetamol.

## Introduction

A vital component of the job performed by pediatric anesthesiologists is the management of pain following surgery. Analgesia via the caudal epidural route is a highly dependable, widely used, and secure procedure that effectively offers pain relief for surgeries below the umbilicus. Performing a caudal injection through the sacral hiatus is quite simple since the injection site is located at a distance from the surgical site [1]. Pain transmission through peripheral nerves induces plasticity in the central nervous system, leading to a heightened and longer sense of pain, even after the painful stimuli have ceased [2]. Preemptive analgesia is given prior to the start of pain to inhibit central nervous system plasticity, resulting in more efficient pain reduction [3].

Nevertheless, the short duration of pain relief after a single injection of local anesthetics necessitates the search for a supplementary substance that can safely extend it. Recently, the inclusion of adjuvants such as opioids, ketamine, and alpha agonists with local anesthetics has led to an extension in the duration of the block, enhanced pain management, increased satisfaction among parents, and quicker recovery [4].

Ropivacaine is a local anesthetic medication that belongs to the family of n-alkyl-substituted pipecoloxylidide, which is an amino-amide. This is a novel, extended-release amide local anesthetic that has fewer toxic effects on the heart and central nervous system. It also offers improved differentiation between sensory and motor effects [5]. Nalbuphine is an opioid belonging to the phenanthrene group that acts as a mixed kappa-agonist and mu-antagonist. Stimulation of spinal and paraspinal opioid receptors results in effective pain relief with minimal side effects such as drowsiness, nausea, vomiting, itching, difficulty urinating, respiratory depression, and steady cardiovascular function [6]. The concurrent use of a local anesthetic with an opioid mitigates the dose-dependent adverse effects associated with either drug alone. The ceiling effect of nalbuphine, as the dose increases, limits its ability to alleviate the most intense pain. However, this action also avoids unwanted sedation and respiratory depression [7].

The study's main objective was to compare the length of time and efficacy of pain relief achieved by administering ropivacaine (Group R) alone versus ropivacaine combined with nalbuphine (Group RN) administered caudally. The secondary objective of the study was to monitor and document negative reactions such as low blood pressure, itching, and difficulty breathing and respond appropriately to address them.

## Materials and methods

After receiving approval from the Institute Ethics Committee (IEC) of All India Institute of Medical Sciences (AIIMS), Raipur, under proposal number AIIMS/RPR/PGTRC(S)/2021/027. This study was prospectively registered in the Clinical Trials Registry-India with registration number CTRI/2023/06/053805. The study was conducted over a period of one and a half years after approval from the IEC (start date of study: 11/03/2022; end date of study: 24/06/2023).

This study was a pilot, randomized, double-blind trial that included children who were scheduled for pediatric infra-umbilical surgeries classified under the American Society of Anesthesiologists (ASA) Grades 1 and 2 in the age category of two to 12 years. The study complies with institutional protocol and has specific exclusion criteria, including parents who decline to give consent, patients with infection over the injection site, patients who have used opioids before a surgical procedure, people who have already undergone lumbar spine surgery, patients having any contraindications to regional anesthesia, patients exhibiting drug hypersensitivity and any abnormality in the lumbosacral region.

Sixty patients admitted for routine infra-umbilical surgical procedures under general anesthesia were enrolled in this double-blind, randomized study. Double blinding was achieved by concealing the drug to the patient and the principal investigator or the one who was collecting the data. Following caudal block under general anesthesia, 30 patients were allocated to each group:

Group R received 0.75 mL/kg - 0.25% ropivacaine diluted in saline 0.9%

Group RN received 0.75 mL/kg - 0.25% ropivacaine + nalbuphine 0.1 mg/kg

Computer-based block randomization was used to randomly divide the patients into two groups (Group R = 30 and Group RN = 31). The random sequences were inserted into opaque, sealed envelopes with sequential numbers to mitigate the influence of any potential confounding variables. The envelope was opened up by a qualified healthcare worker (HCW) who was not part of the study. The HCW then prepared the study drugs as per the group allocation and handed them over to the anesthesiologist performing the procedure, and the person collecting data and providing postoperative care was blinded to group allocation.

Patients who met the inclusion criteria underwent a period of fasting for six to eight hours for solid food and two hours for clear liquids before surgery. Following the patient's informed written consent, they were escorted to the operating room. Standard ASA monitors were connected to the patient, and baseline data were documented. Intravenous (IV) access was established. Anesthesia was initiated with the analgesic fentanyl (2 mcg/kg), the induction agent propofol (2 mg/kg), and the muscle relaxant atracurium (0.5 mg/kg) to aid in endotracheal intubation. Once the airway was secured, the patients' lungs were ventilated mechanically using a mixture of nitrous oxide and oxygen (50%:50%). The end-tidal CO2 level was maintained between 30 and 35 mmHg. The anesthesia was maintained using sevoflurane at a minimum alveolar concentration for the given age (MACage) of 1-1.2 [8]. After repositioning the patients on their side, under all aseptic precautions using the landmark-guided technique, a caudal epidural block was performed 20 minutes before the surgical incision.

Group R was administered a dose of 0.75 mL/kg of body weight of a 0.25% solution of ropivacaine. Group RN was administered a dosage of 0.75 mL/kg of 0.25% ropivacaine together with 0.1 mL/kg of nalbuphine. Heart rate (HR), non-invasive blood pressure (NIBP), end-tidal carbon dioxide (EtCO2), oxygen saturation (SPO2), and peripheral temperature (axillary site) were continuously monitored during the procedure. Any adverse events that occurred were documented and addressed. Additional doses of atracurium were administered after evaluating the neuromuscular function using the train of four methods. If there is an HR or BP increase of more than 20%, a bolus of 0.5 mcg/kg of fentanyl will be administered for rescue analgesia and controlled accordingly. The total number of administered fentanyl dosages was observed and documented. Following the surgical procedure, the patient was subjected to the reversal process and subsequently extubated in accordance with the established protocol. Hemodynamic parameters were constantly monitored in the postoperative period. Pain levels were assessed using the Face, Legs, Activity, Cry, and Consolability (FLACC) scale every hour for the first four hours, then every two hours for the following eight hours, and finally, every four hours for the next 12 hours. Episodes of hypotension (a 20% reduction in mean arterial pressure compared to baseline values), bradycardia (HR below 50 beats per minute (BPM)), or hypoxemia (oxygen saturation below 90%) were documented and treated appropriately. After the operation, IV paracetamol (PCM) was administered every eight hours to both groups if the FLACC score was greater than two. The dosage of PCM was 15 mg/kg of body weight.

Statistical analysis

Data entry was carried out using statistical packages for SPSS 16.0 for Windows (IBM SPSS Statistics for Windows, IBM Corp., Armonk, NY). The chi-square test was used to compare categorical variables. The Shapiro-Wilk test was employed to analyze continuous data, and the results were reported as the mean and standard deviation (SD). The Wilcoxon-Mann-Whitney test was employed to analyze data that did not follow a normal distribution. The Friedman test was employed to assess the variations within the group over time. The generalized estimating equations approach was employed to investigate the disparity in temporal changes of data between two groups. p-values were computed using the log-rank test for categorical variables and Cox-proportional hazard (PH) regression for continuous variables. A p-value less than 0.05 was considered statistically significant.

## Results

**Figure 1:**
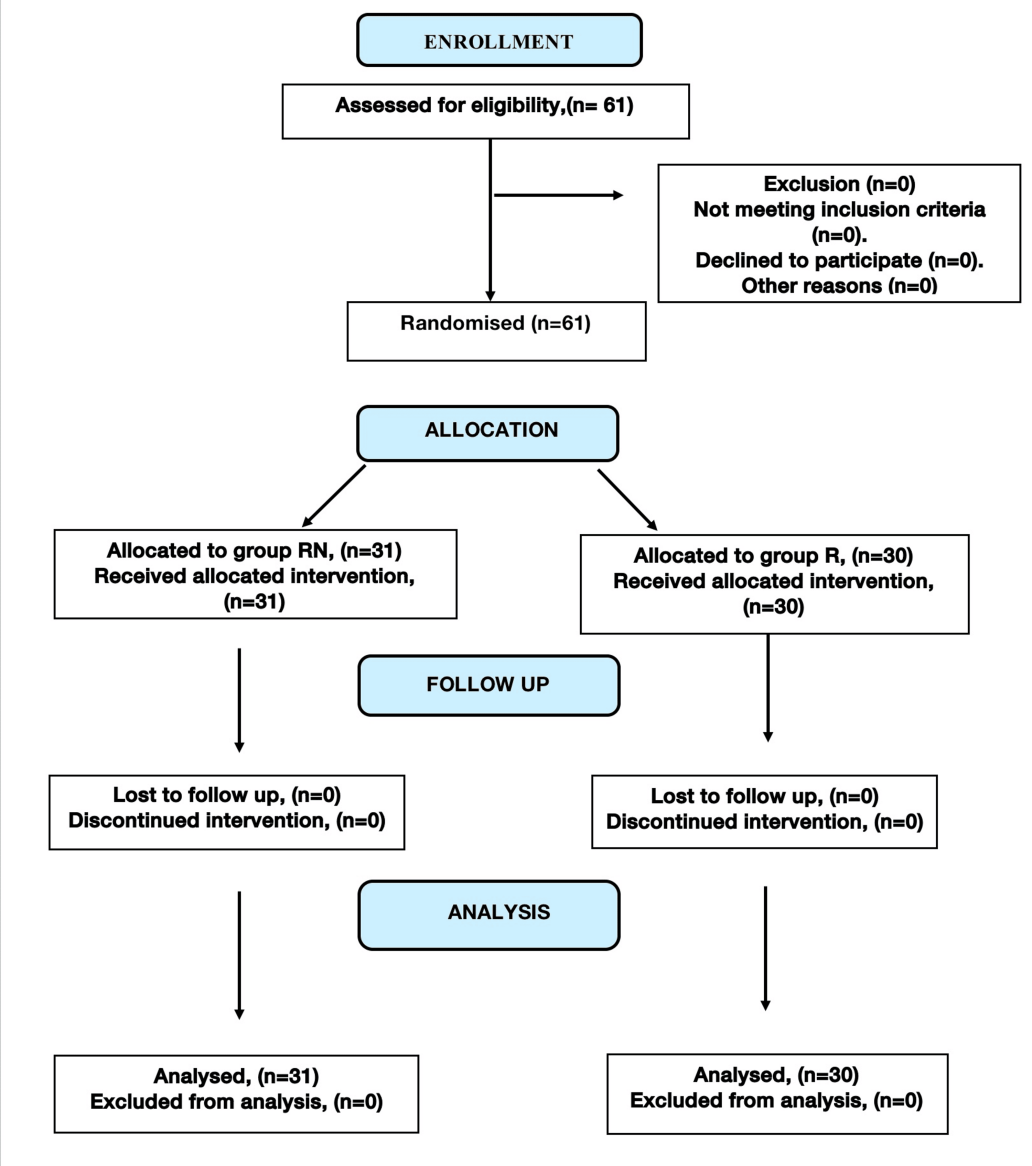
Consolidated Standards of Reporting Trials (CONSORT) diagram for the study

A total of 61 participants were included in the study (Figure 1).

**Figure 2:**
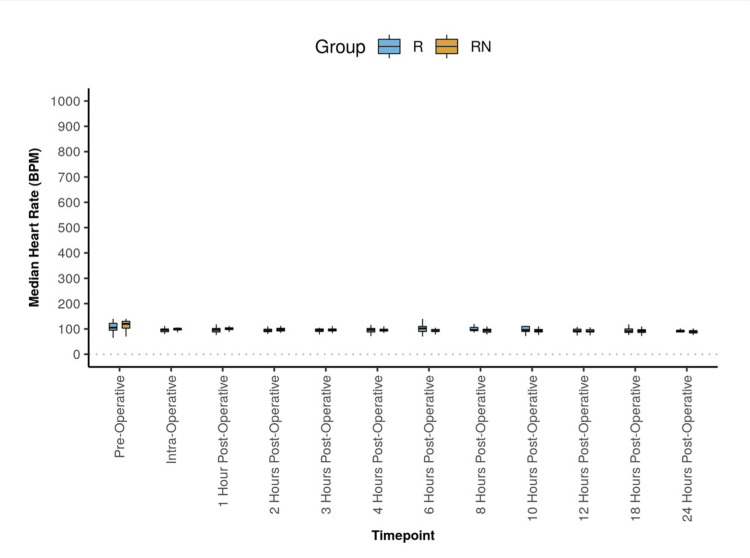
Figure denoting the change in mean heart rate at different time points in both groups The Box-and-Whisker plot depicts the distribution of heart rate (BPM) over different time points. In each box, the middle horizontal line represents the median heart rate (BPM), the upper and lower bounds of the box represent the 75th and the 25th centile of heart rate (BPM), respectively, and the upper and lower extent of the whiskers represent the Tukey limits for heart rate (BPM) at each of the time points, respectively.

Demographic parameters like age, gender, ASA, and duration of surgery were comparable between the two groups (Table 1). The two groups differed significantly in HR (BPM) at the following time points: intra-operative and one-hour postoperative. In Group R, the mean HR increased from 108.55 preoperatively to a maximum of 131.76 at six hours postoperatively. It then decreased to 92.00 at the 24-hour postoperative period. The difference was statistically significant. (Friedman test: χ^2^ = 34.5, p < 0.001). The mean HR in Group RN gradually went down from 114.87 in the preoperative time point to 90.71 in the 24-hour postoperative period. This difference was statistically significant (Friedman test: χ^2^ = 93.6, p < 0.001) (Figure 2).

**Table 1:** Demographics showing age distribution, gender, ASA status, and duration of surgery in both groups ASA, American Society of Anesthesiologists

Details	Mean ± SD | median (IQR) | min-max OR N (%)
Group	
R	30 (49.2%)
RN	31 (50.8%)
Age (years)	4.93 ± 2.60
Age	
Two to five years	44 (72.1%)
Six to 12 years	17 (27.9%)
Gender	
Male	52 (85.2%)
Female	9 (14.8%)
ASA	
I	58 (96.7%)
II	2 (3.3%)
Duration of surgery (minutes)	83.77 ± 35.21

**Figure 3:**
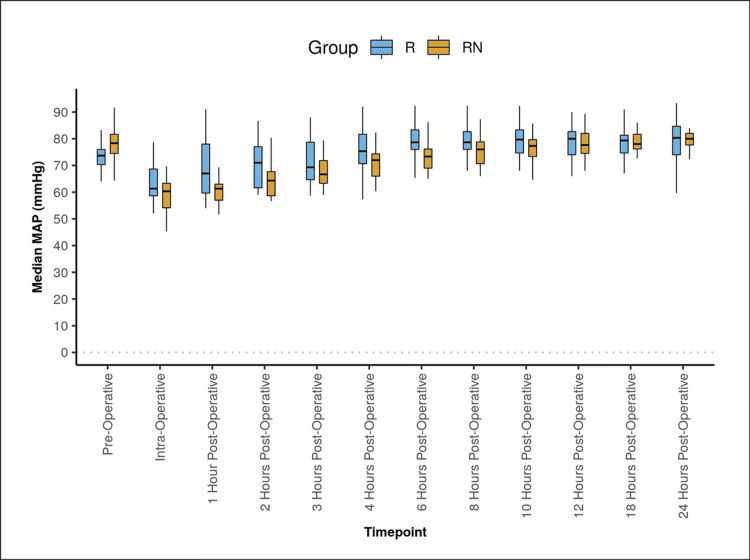
Mean arterial pressure (MAP) at different time points in both groups The Box-and-Whisker plot depicts the distribution of mean arterial pressure (MAP) (mmHg) over different time points. In each box, the middle horizontal line represents the median MAP, the upper and lower bounds of the box represent the 75th and the 25th centile of MAP, respectively, and the upper and lower extent of the whiskers represent the Tukey limits for MAP at each of the time points, respectively.

Both groups differed significantly in terms of MAP (mmHg) at the following time points: pre-operative, one hour postoperative, two hours postoperative, four hours postoperative, six hours postoperative, and eight hours postoperative. This difference was found to be statistically significant (Figure 3). Both groups differed significantly in terms of FLACC score at various time points in the postoperative period.

In Group R, the mean FLACC score increased from zero at the one-hour postoperative to a maximum of two at the six hours postoperatively and then decreased to 0.66 (0) at the 24 hours postoperatively. This change was statistically significant (Friedman test: χ^2^ = 137.0, p < 0.001).

**Figure 4:**
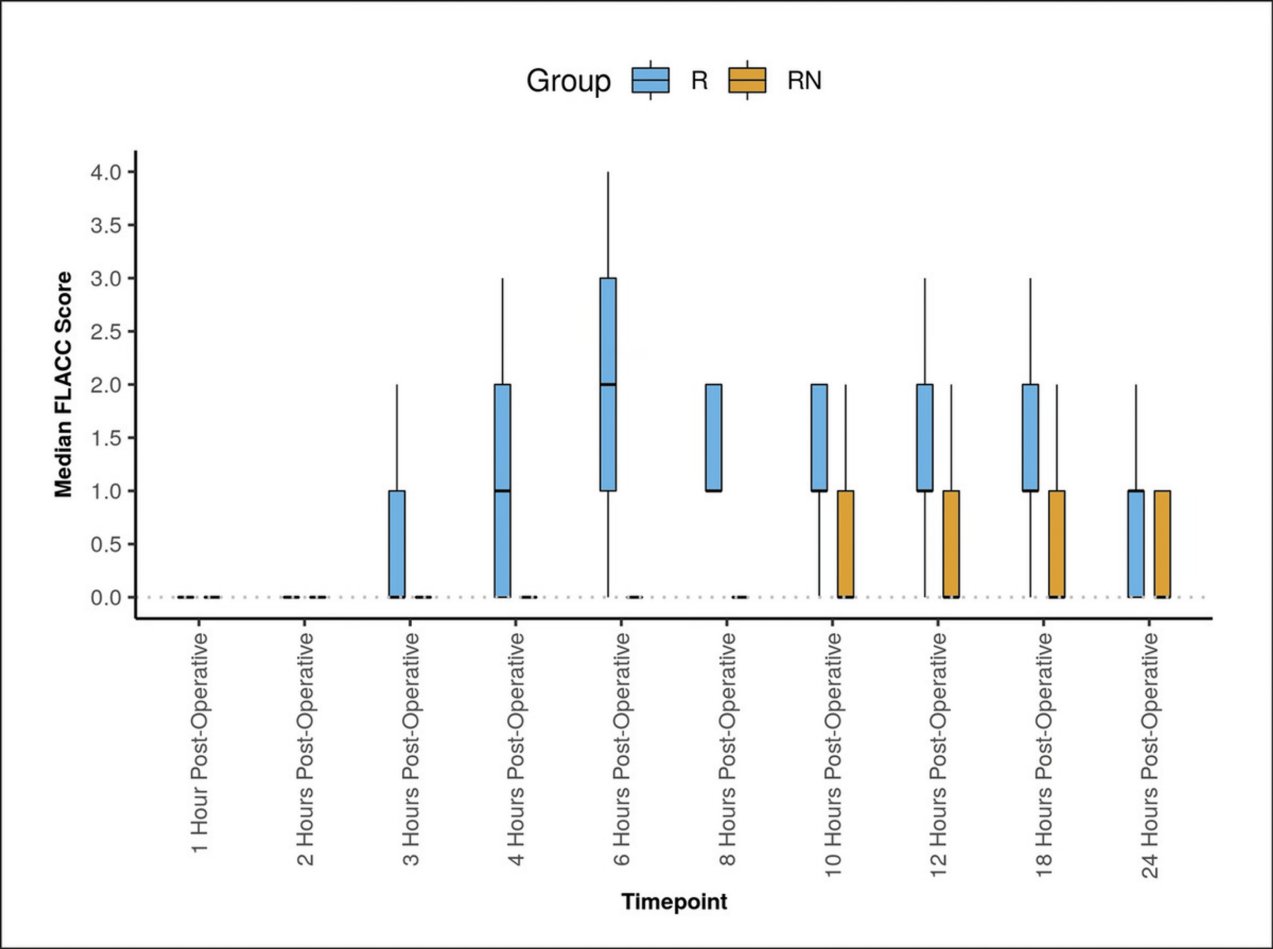
Figure denoting the FLACC scale score in both the groups The Box-and-Whisker plot depicts the distribution of the FLACC score over different time points. In each box, the middle horizontal line represents the median FLACC score, the upper and lower bounds of the box represent the 75th and the 25th centile of the FLACC score, respectively, and the upper and lower extent of the whiskers represent the Tukey limits for FLACC score at each of the time points, respectively. FLACC, Face, Legs, Activity, Cry, and Consolability

In Group RN, the mean FLACC Score increased from zero at the one-hour postoperative period to a maximum of 0.61 at the 10 hours postoperatively, then decreased to 0.29 at the 24-hour postoperatively. The change was statistically significant (Friedman test: χ^2^ = 63.3, p < 0.001). There was a significant difference in FLACC score between the two groups (p < 0.001) over time (Figure 4).

There was no significant difference in the distribution of intra-operative additional fentanyl requirement (χ^2^ = -, p = -); in 100.0% of the participants in both groups, no intra-operative additional fentanyl was required (Table 2). The mean (SD) time to the first PCM (hours) in Group R was 7.07 (1.85). In the group, the RN was 13.14 (4.99).

**Table 2:** Information about the requirement for additional intraoperative fentanyl

Intra-operative additional fentanyl required	Group		
	R	RN	Total
No	30 (100.0%)	31 (100.0%)	61 (100.0%)
Total	30 (100.0%)	31 (100.0%)	61 (100.0%)

The median (IQR) of time to first PCM (hours) in Group R was 7 (6-8), and in the RN group was 11 (10-17.75). Time to first PCM in Group R ranged from five to 12 hours, and in Group RN, it ranged from six to 24 hours. A significant difference was observed between the two groups (W = 37.000, p < 0.001), with the median time being highest in Group RN (Table 3). There was a significant difference between the various groups regarding the distribution of postoperative additional PCM required (χ^2^ = 22.191, p < 0.001).

**Table 3:** Time to first dose of paracetamol between the two groups

Time to first PCM (hours)	Group		Wilcoxon-Mann-Whitney U test	
	R	RN	W	p-value
Mean (SD)	7.07 (1.82)	13.14 (4.99)	38	<0.001
Median (IQR)	7 (6-8)	11 (10-17.75)		
Min-max	5-12	6-24		

The strength of association between the two variables (Cramér's V) was 0.61 (high association). The strength of association between the two variables (Bias et al.'s V) was 0.6 (high association); 100.0% of the participants in Group R required postoperative additional PCM. However, only 45.2% of the participants in Group RN required postoperative additional PCM (Table 4).

**Table 4:** Postoperative additional intravenous paracetamol requirement

Postoperative additional PCM required	Group			Chi-squared test	
	R	RN	Total	χ^2^	p-value
Yes	30 (100.0%)	14 (45.2%)	44 (72.1%)	22.808	<0.001
No	0 (0.0%)	17 (54.8%)	17 (27.9%)		
Total	30 (100.0%)	31 (100.0%)	61 (100.0%)		

## Discussion

Analgesia via the caudal epidural route is one of the safest treatments used to control both intraoperative and postoperative pain in children. It is relatively secure and has an enhanced level of protection [9]. Children exhibit heightened pain sensitivity, and the challenge lies in their ability to effectively communicate their distress [10]. In addition to the surgical element, pain itself stimulates inflammatory responses that hinder the healing of wounds. Therefore, sufficient pain management is crucial not only for alleviating pain but also for enhancing recovery [11].

This study compared the duration of postoperative analgesia in children aged two to 12 years undergoing infra-umbilical surgeries under general anesthesia. The study used plain ropivacaine and nalbuphine as an additive in caudal epidural. The results showed that the duration of postoperative analgesia was significantly longer in the nalbuphine group (24 hours) compared to the control group. The essential parameters of HR, systolic blood pressure (SBP), and diastolic blood pressure (DBP) were evaluated. There was a notable decrease in all vital signs throughout the surgery in both groups. However, in the postoperative phase, the RN group exhibited stable vital signs for the entire 24-hour duration. In group R, the postoperative vitals experienced a modest increase after six hours; however, this was alleviated by administering PCM. During the surgery, the need for inhalation anesthetic drugs was reduced, and there was no need for supplemental opioids except during the induction phase. There was no notable alteration in oxygen saturation or respiratory rate between the two groups after the operation. The SpO2 readings consistently ranged from 99% to 100% when the patient was breathing normal room air, and the respiratory rate remained consistent with the patient's baseline values throughout the postoperative period.

The FLACC scoring in both groups did not exceed a score of three, indicating that there is no need for postoperative administration of fentanyl. PCM was required in Group R for postoperative use in nearly all children at a frequency of every six hours. However, the pain was effectively managed with PCM. Within group RN, a notable decrease in the need for postoperative analgesics was seen. In 45% of the children, there was no need for postoperative pain relief for a period of 24 hours. In 55% of cases, a single dose of PCM was sufficient, and the duration of the dose exceeded 10 hours, which is notably longer than in Group R. None of the patients experienced any adverse effects, such as bradycardia, hypotension, or respiratory depression, after the operation. Additionally, there were no severe adverse effects observed in any of the patients, such as respiratory depression or gastrointestinal issues, from the use of nalbuphine.

This study is the first to assess pain relief after surgery using ropivacaine with nalbuphine as an adjuvant in the caudal epidural. A study conducted by Doctor et al. [12] compared the effects of caudal ropivacaine (0.2%) and bupivacaine (0.25%) with fentanyl (1 mcg/kg). The study found that fentanyl did not provide any additional benefits at a lower dose, and at a larger level, it caused systemic side effects. The study concluded that ropivacaine was a superior choice owing to its less powerful motor block. Our investigation found that the use of ropivacaine resulted in minimal motor blockage and no side effects.

Elfawal et al. [13] did a study comparing the use of caudal bupivacaine (0.125%, 1 mL/kg) with nalbuphine (0.2 mg/kg) to fentanyl (1 mcg/kg) in a 2 mL solution. The study focused on pediatric patients aged two to six years who were scheduled for inguinal hernia operations. The study revealed that nalbuphine offered a longer duration of pain relief (24 hours) without the need for additional pain medications, but fentanyl provided a shorter duration of pain relief (9.1 to 7.8 hours) with a higher consumption of analgesics (236 ± 248.9 mg/24 hours). Regarding the negative consequences, four patients who were administered nalbuphine experienced vomiting, while one patient in the nalbuphine group and three patients in the fentanyl group developed itching. However, in our investigation, the dosage of nalbuphine was only 0.1 mg/kg. The results were consistent with the current study on postoperative analgesia, and no side effects were observed. This may be attributed to the lower dosage of nalbuphine.

A study conducted by Salama et al. [14] compared the use of plain levobupivacaine-L (0.125% 1 mL/kg) to levobupivacaine with nalbuphine-LN (0.2 mg/kg) in pediatric caudal epidural procedures. The nalbuphine group experienced postoperative analgesia lasting six to eight hours, while the plain levobupivacaine group had analgesia lasting two to three hours. The postoperative follow-up duration in this study was limited to only 12 hours. However, our study had a postoperative follow-up period of 24 hours, during which the dose of nalbuphine administered was 0.1 mg/kg. The effects of the medication lasted until 24 hours after the surgery. Decreasing the concentration of levobupivacaine may result in a shorter duration of its effects.

Limitations

Since the caudal epidural we utilized is a blind procedure, there is a possibility of subcutaneous injection of the medicine if the epidural space is not well confirmed. The sacral vertebrae present anatomical challenges due to many forms of vertebral abnormalities, as well as the advancing age of the infant.

## Conclusions

To conclude, nalbuphine as an additive to ropivacaine administered through the caudal epidural route provided better analgesia and prolonged the duration of analgesia postoperatively with decreased use of postoperative IV PCM. The overall requirement of PCM has drastically decreased with the use of nalbuphine and even offered some sedation in the child without any adverse effects.

## Figures and Tables

**Figure 5:**
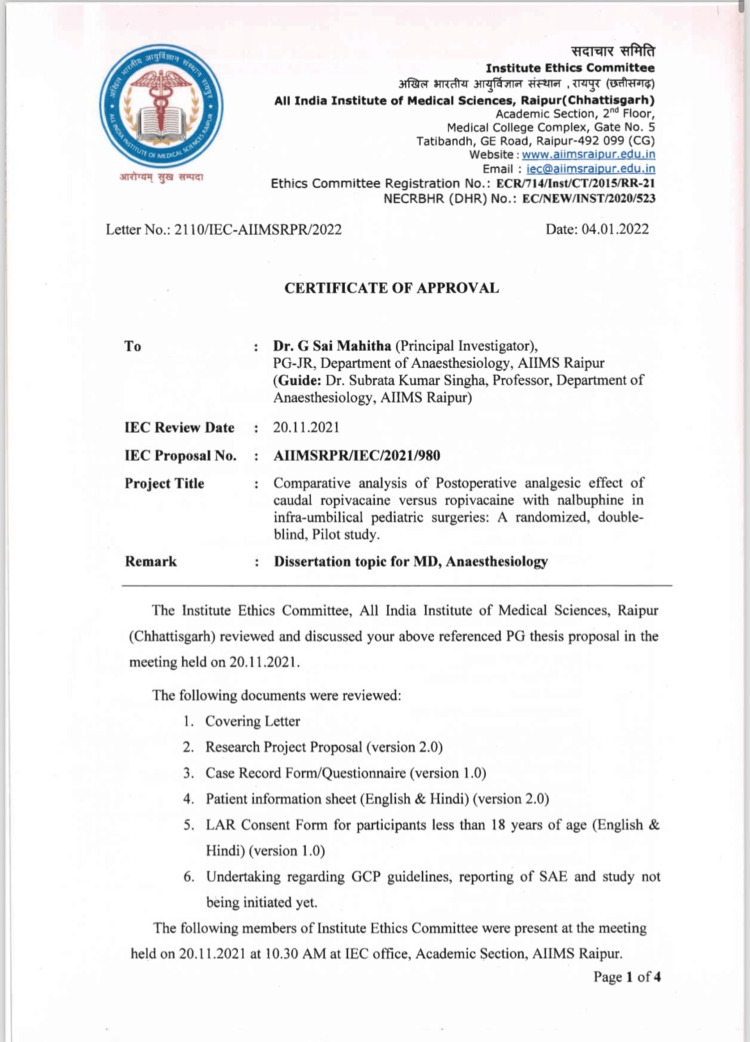
IEC approval letter

**Figure 6:**
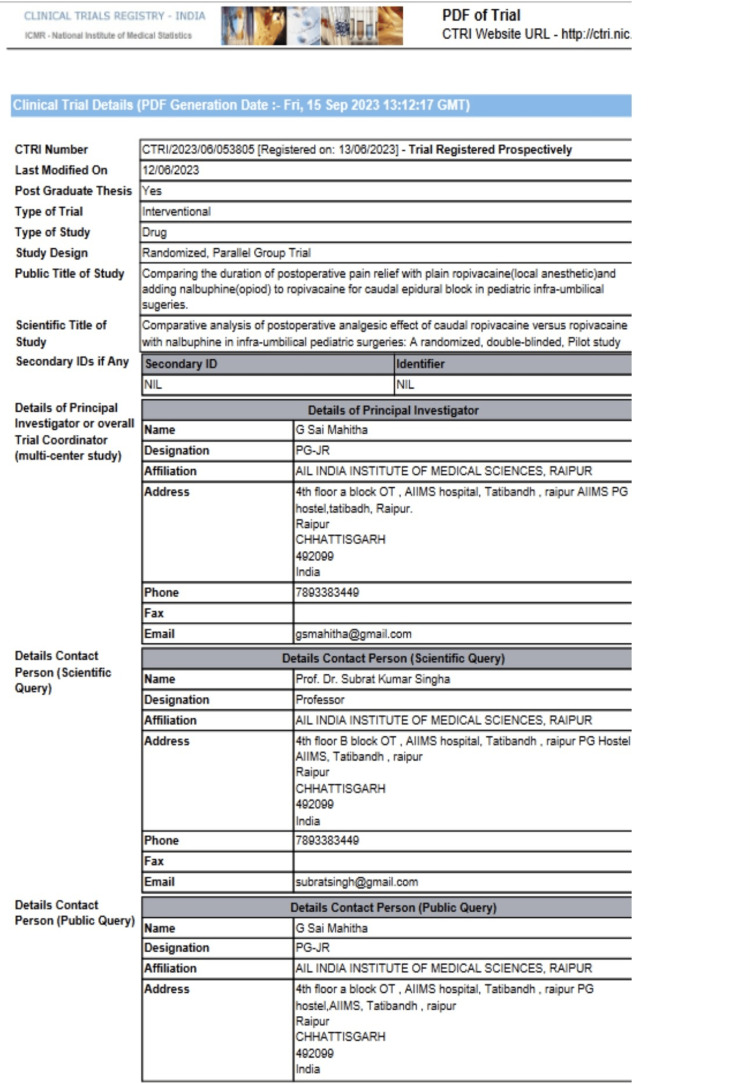
CTRI approval letter
